# Immunogenicity, Reactogenicity, and Safety of AS01_E_-adjuvanted Respiratory Syncytial Virus (RSV) Prefusion F Protein-based Candidate Vaccine (RSVPreF3 OA) When Co-administered With a Seasonal Quadrivalent Influenza Vaccine in Older Adults: Results of a Phase 3, Open-Label, Randomized Controlled Trial

**DOI:** 10.1093/cid/ciad786

**Published:** 2024-01-08

**Authors:** Reynaldo Chandler, Nathali Montenegro, Cecilia Llorach, Lorena Noriega Aguirre, Sophie Germain, Sherine O Kuriyakose, Axel Lambert, Dominique Descamps, Aurélie Olivier, Veronica Hulstrøm

**Affiliations:** CAENSA Clinical Trials, Panamá City, Panamá; Centro de Vacunación e Investigación CEVAXIN S.A., Panamá City, Panamá; Unidad Local de Atención Primaria de Salud de San Cristóbal, Caja de Seguro Social, Panamá; Instituto de Investigaciones Científicas y Servicios de Alta Tecnología AIP (INDICASAT AIP), Panamá City, Panamá; Centro de diagnóstico y tratamiento de enfermedades respiratorias, CEDITER, Panamá City, Panamá; GSK, Wavre, Belgium; GSK, Bangalore, India; GSK, Wavre, Belgium; GSK, Wavre, Belgium; GSK, Wavre, Belgium; GSK, Wavre, Belgium

**Keywords:** respiratory syncytial virus, AS01_E_-adjuvanted RSV prefusion F protein–based candidate vaccine, influenza, co-administration, investigational vaccine

## Abstract

**Background:**

Co-administration of vaccines against respiratory syncytial virus (RSV) and influenza can be considered given their overlapping seasonality and may increase vaccine uptake and compliance. In this phase 3, open-label, randomized study, we evaluated the immunogenicity, reactogenicity, and safety of the AS01_E_-adjuvanted RSV prefusion F protein–based candidate vaccine (RSVPreF3 OA) when co-administered with a seasonal quadrivalent influenza vaccine (FLU-QIV) in older adults.

**Methods:**

Participants aged ≥60 years (randomized 1:1) received either RSVPreF3 OA and FLU-QIV simultaneously on day 1 (Co-Ad group) or FLU-QIV on day 1 followed by RSVPreF3 OA on day 31 (sequential administration [SA] group). The co-primary objectives were to demonstrate noninferiority of RSVPreF3 OA in terms of RSV-A neutralization geometric mean titer (GMT) ratio and FLU-QIV in terms of hemagglutination inhibition GMT ratio for each FLU-QIV strain, when co-administered versus when administered alone at 1 month post-vaccination. Noninferiority was demonstrated if the upper limit of the 95% confidence interval of the group GMT ratio (SA/Co-Ad) was ≤1.5. Secondary descriptive objectives comprised additional immunogenicity assessments, reactogenicity, and safety.

**Results:**

Of the 885 participants who received 1 dose of the study vaccines, 837 were included in the per protocol set. Demographic and baseline characteristics were balanced between the groups. Both co-primary objectives were met for both vaccines. Reported adverse events in both groups were mild to moderate, with a low frequency of grade 3 events.

**Conclusions:**

Data from this study demonstrate that RSVPreF3 OA can be co-administered with FLU-QIV. Co-administration is well tolerated, with an acceptable safety profile.

**Clinical Trials Registration.** NCT04841577.

## Teaser

This study supports co-administration of the AS01_E_-adjuvanted respiratory syncytial virus prefusion F protein–based candidate vaccine (RSVPreF3 OA) and a seasonal quadrivalent influenza vaccine in older adults. Co-administration demonstrated an acceptable immunogenicity and safety profile.

Respiratory syncytial virus (RSV) is a leading pathogen causing lower respiratory disease in young children [1–3] and older adults [4–7]. A weakened immune system, due to aging and comorbidities, underpins a high RSV disease burden in older adults [8, 9]. In a US study involving 608 healthy individuals aged ≥65 years and 540 high-risk patients with cardiopulmonary disease (over a 4-year period), RSV infection was identified in 3%–7% and 4%–10% of healthy and high-risk participants, respectively [4]. A systematic literature review and meta-analysis of 21 studies in high-income countries estimated that in adults aged ≥60 years, RSV-associated acute respiratory infections in 2019 would approximate 5.2 million cases, with 470 000 hospitalizations and 33 000 in-hospital deaths [10]. Despite this, and unlike influenza, which is a well-documented cause of severe morbidity and mortality in older adults, RSV remains clinically under-recognized as a cause of respiratory disease in adults [11]. However, both RSV and influenza are associated with clinical and economic burden [12–14].

Countries with a temperate climate mostly experience fall–winter RSV epidemics [15, 16], and RSV annual seasonality patterns largely overlap with those of influenza [16]. In a prospective epidemiologic study in 14 countries across North America, Europe, and East Asia, on average, 18.7% and 7.4% of the moderate-to-severe influenza-like episodes in adults aged ≥65 years were attributed to influenza A and RSV, respectively. However, despite infection rates being higher with other viral respiratory pathogens, RSV-positive moderate-to-severe illnesses were more likely to lead to hospitalization. Notably, hospitalization was 5-fold more common among those with RSV-positive moderate-to-severe illness compared with influenza A [5]. In their study of hospitalized adults aged ≥60 years, Ackerson et al. found that RSV infection may result in greater morbidity and mortality than influenza, with a potentially greater impact on long-term survival [11].

Several candidate vaccines are in development [17–23]; based on the viral envelope F glycoprotein, responsible for membrane fusion and host cell entry, as it is highly conserved across RSV subgroups [24] and induces a neutralizing antibody response [25, 26]. The recombinant subunit RSV vaccine, AS01_E_-adjuvanted RSV prefusion F protein–based candidate vaccine (RSVPreF3 OA), based on the RSV prefusion F glycoprotein (RSVPreF3) of the RSV-A2 strain [17] and the adjuvant system AS01_E_ has shown efficacy in preventing RSV-induced lower respiratory tract disease and the full spectrum of RSV diseases, exhibiting a robust immune response and an acceptable safety profile and tolerability [27–31]. Based on this, RSVPreF3 OA was recently approved by the US Food and Drug Administration and the European Medicines Agency for the prevention of lower respiratory tract disease caused by RSV in individuals aged ≥60 years [32, 33].

Routine, annual seasonal influenza vaccination is recommended by the US Advisory Committee on Immunization Practices for individuals aged ≥6 months without contraindications [34]. It is also recommended by the European Centre for Disease Prevention and Control for individuals according to member country guidelines, particularly for high-risk populations, such as older adults [35], and by the World Health Organization for high-priority groups, including health workers and older adults [36]. Given the overlapping seasonality of RSV with influenza infections [16], co-administration of RSV and influenza vaccines could present the benefits of promoting vaccine acceptance and uptake and achieving protection against both infections with reduced healthcare visits [37, 38].

This study aimed to assess the noninferiority of the elicited humoral immune response, and the safety and reactogenicity profile of RSVPreF3 OA vaccine when co-administered with seasonal quadrivalent influenza vaccine (FLU-QIV) in adults aged ≥60 years, compared with a sequential administration (SA) schedule.

## METHODS

### Study Design

**Figure 1.**
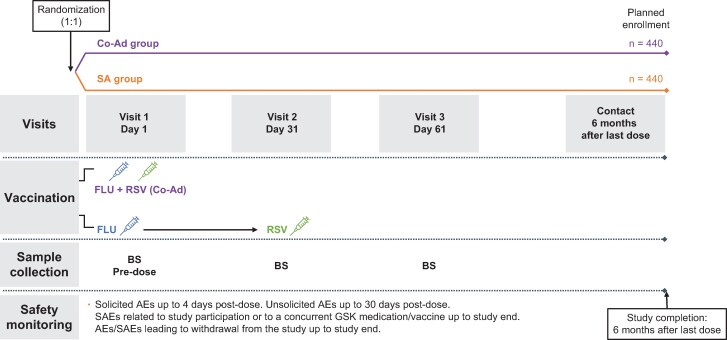
Study design. Abbreviations: AE, adverse event; BS, blood sample; Co-Ad, co-administration; RSV, respiratory syncytial virus; SA, sequential administration; SAE, serious adverse event.

This phase 3, open-label, randomized study (NCT04841577) was conducted at 14 centers in 3 countries: 7 in New Zealand, 5 in Panama, and 2 in South Africa. Participants were randomized (1:1) to 2 parallel groups: the co-administration (Co-Ad) group received 1 dose of RSVPreF3 OA vaccine and 1 dose of FLU-QIV at visit 1 (day 1); the SA group received 1 dose of FLU-QIV at visit 1 (day 1), followed by 1 dose of RSVPreF3 OA vaccine at visit 2 (day 31) (Figure 1). See Supplementary Methods for randomization methods.

This study was conducted in accordance with the ethical principles that have their origins in the Declaration of Helsinki and the principles of Good Clinical Practice and was approved by all applicable regulatory agencies, investigational center ethics committees, or institutional review boards.

### Participants

Participants were individuals aged ≥60 years at time of first study intervention who, in the investigators’ opinion, could and would comply with protocol requirements, and who provided informed consent before any study-specific procedure. Participants were living in the general community or in assisted-living facilities that provided minimal assistance, such that the participant was primarily responsible for self-care and activities of daily living. Individuals with chronic medical conditions, with/without specific treatment, were included in the study if considered medically stable by the investigator. See Supplementary Methods for exclusion criteria.

### Vaccine Composition, Dosage, and Route of Administration

Single dose (0.5 mL) RSVPreF3 OA vaccine (GSK3844766A, GSK Vaccines) containing 120 µg of recombinant RSVPreF3 antigen and AS01_E_ adjuvant was prepared and administered by intramuscular injection in the deltoid of the non-dominant arm. The AS01_E_ adjuvant is a liposome-based adjuvant system consisting of 25 μg 3-O-desacyl-4′-monophosphoryl lipid A and 25 μg QS-21 (Quillaja saponaria Molina, fraction 21).

Single dose (0.5 mL) FLU-QIV (Fluarix Quadrivalent or Fluarix Tetra, GSK) containing 15 µg hemagglutinin per strain/dose was prepared and administered by intramuscular injection in the deltoid of the dominant (Co-Ad group) or the non-dominant (SA group) arm (Figure 1). The 4 influenza strains included in FLU-QIV were: Flu A/Hong Kong/2671/2019 H3N2 (FLU A/H3N2), Flu A/Victoria/2570/2019 H1N1 (FLU A/H1N1), Flu B/Phuket/3073/2013 Yamagata (FLU B/Yamagata), and Flu B/Washington/02/2019 Victoria (FLU B/Victoria).

### Outcomes/Assessments

Co-primary objectives were to demonstrate noninferiority of RSVPreF3 OA in terms of RSV-A neutralization geometric mean titer (GMT) ratio and FLU-QIV in terms of hemagglutination inhibition (HI) GMT ratio for each FLU-QIV strain when co-administered versus when administered alone at 1-month post-vaccination. Secondary immunogenicity objectives included further evaluation of the immune response 1 month post-RSVPreF3 OA vaccination in terms of RSV-A neutralization titers expressed as mean geometric increase (MGI) and, in a subset of participants, RSV-B neutralization titers expressed as group GMT ratio and MGI. HI titers expressed as MGI, as well as HI seroconversion rate (SCR) and seroprotection rate (SPR) for each FLU-QIV strain, were assessed 1 month after the FLU-QIV dose. The Center for Biologics Evaluation and Research (CBER) and the Committee for Medicinal Products for Human Use (CHMP) criteria for influenza vaccines were evaluated [39, 40]. Blood samples were collected from participants in the Co-Ad and SA groups at pre-vaccination (day 1) and 1 month post-vaccination (day 31). An additional blood sample was collected in the SA group 1 month post-RSVPreF3 OA vaccination (day 61) (Figure 1). Details on clinical laboratory tests (RSV-A and RSV-B neutralization assays) are in Supplementary Methods.

Secondary safety and reactogenicity objectives included: solicited adverse events (AEs; local: erythema, pain, swelling; and systemic: arthralgia, fatigue, fever, headache, myalgia) with onset within 4 days (ie, day of vaccination and the 3 subsequent days) after vaccine administration; unsolicited AEs (including potential immune-mediated diseases [pIMDs], non-serious AEs, or serious AEs [SAEs]) within 30 days after vaccine administration (ie, day of vaccination and the 29 subsequent days). Solicited and unsolicited AEs were noted on a participant paper diary card.

### Statistical Analyses

The study planned to enrol 880 participants (see Supplementary Methods for sample size calculation). Participants who withdrew/were lost to follow-up were not replaced; missing data were not imputed. Two analysis sets were used: exposed set (ES) and per protocol set (PPS). The analyses per group were based on the study intervention administered.

The primary analyses for co-primary and secondary immunogenicity endpoints were performed in the PPS, which included all eligible participants who received ≥1 study intervention as per protocol, had immunogenicity results pre-/post-dose for ≥1 antigen, and complied with blood draw intervals. Contribution of participants to the PPS at any given timepoint was defined by timepoint, without intercurrent medical conditions that may interfere with immunogenicity and without prohibited concomitant medication/vaccination. For co-primary objectives, noninferiority was demonstrated if the upper limit (UL) of the 95% confidence interval (CI) of the group GMT ratio (SA/Co-Ad) was ≤1.5 for the RSVPreF3 OA vaccine and each FLU-QIV strain. The co-primary endpoint on RSVPreF3 OA was assessed based only on RSV-A neutralization titers. Multiplicity adjustment was applied using a nominal alpha of 2.5% for each of them to ensure the global type I error for the co-primary objectives is controlled below 2.5%. The 2-sided 95% CI for group GMT ratio was derived from an analysis of covariance (ANCOVA) model, including treatment group, age group at vaccination (aged 60–69, 70–79, or ≥80 years), country, and sex as fixed effects, and the pre-dose log_10_-transformed titer as a covariate. All between-group analyses of secondary endpoints were descriptive, and no type I error adjustment was made for multiplicity of these endpoints.

Safety and reactogenicity endpoints were analyzed on the ES, consisting of participants who received a study intervention. The Medical Dictionary for Regulatory Activities v24.1 was used for safety and reactogenicity analyses on the ES. Statistical analyses used the Statistical Analysis Systems Drug Development software v9.4.

## RESULTS

### Participant Disposition

**Figure 2.**
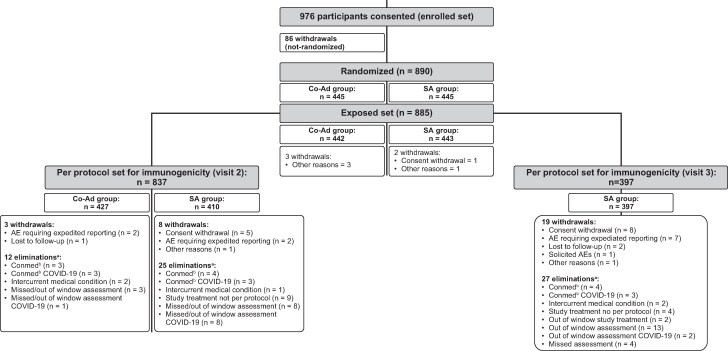
Flow chart of participant enrollment, group allocation, and elimination/exclusion. Abbreviations: AE, adverse event; Co-Ad, co-administration; conmed, concomitant medication; COVID-19, coronavirus disease 2019; PPS, per protocol set; SA, sequential administration. ^a^Participant may have been eliminated from analysis set for more than 1 reason. ^b^Excluded medication, vaccine, or device and administration of any medication forbidden by protocol. In the PPS of the end-of-study analysis, an additional participant in the SA group was eliminated compared with the PPS of the final analysis.

In total, 976 participants were enrolled between 27 April 2021 and 8 February 2022; 890 were randomized 1:1 to Co-Ad or SA (Figure 2). Overall, 885 participants received 1 dose of the study intervention and were included in the ES. Of these, 837 (94.6%) were included in the PPS for visit 2 (Co-Ad, n = 427; SA, n = 410) and 397 (89.6%) were included in the PPS for visit 3 (SA group only). Overall, 39 participants withdrew from the study (Co-Ad, n = 13; SA, n = 26); the main reasons being loss to follow-up (n = 15) and AEs (n = 14; including coronavirus disease 2019 [COVID-19] pneumonia/COVID-19/suspected COVID-19 [n = 5], death [n = 2], and diffuse large B-cell lymphoma, cardiac failure congestive, acute disseminated encephalomyelitis, anemia, subarachnoid hemorrhage, pulmonary embolism, and pancreatic carcinoma [all n = 1]).

Demographic and baseline characteristics were similar between the groups. Mean participant age was 68.4 and 68.6 years in the Co-Ad and SA groups, respectively. Distribution across age categories did not differ between groups (Table 1 [ES]; Supplementary Table 1 [PPS]).

**Table 1.** Summary of Participant Characteristics—Exposed Set

	Co-Ad Group (n = 442)		SA Group (n = 443)	
Characteristic	Value or n	%	Value or n	%
Age at first vaccination				
Mean (SD), y	68.4 (6.9)	…	68.6 (6.9)	…
Age group, y				
60–69	260	58.8	259	58.5
70–79	144	32.6	144	32.5
≥ 80	38	8.6	40	9.0
Sex				
Male	214	48.4	215	48.5
Female	228	51.6	228	51.5
Country				
New Zealand	145	32.8	145	32.7
Panama	153	34.6	157	35.4
South Africa	144	32.6	141	31.8
Race				
Asian	4	0.9	5	1.1
Black	72	16.3	70	15.8
Native Hawaiian or other Pacific Islander	2	0.5	1	0.2
White	137	31	135	30.5
Māori	7	1.6	5	1.1
Mixed race	218	49.3	227	51.2
Other	2	0.5	0	0

Abbreviations: Co-Ad, co-administration; n, number of participants; SA, sequential administration; SD, standard deviation.

### Co-primary Outcomes

**Figure 3.**
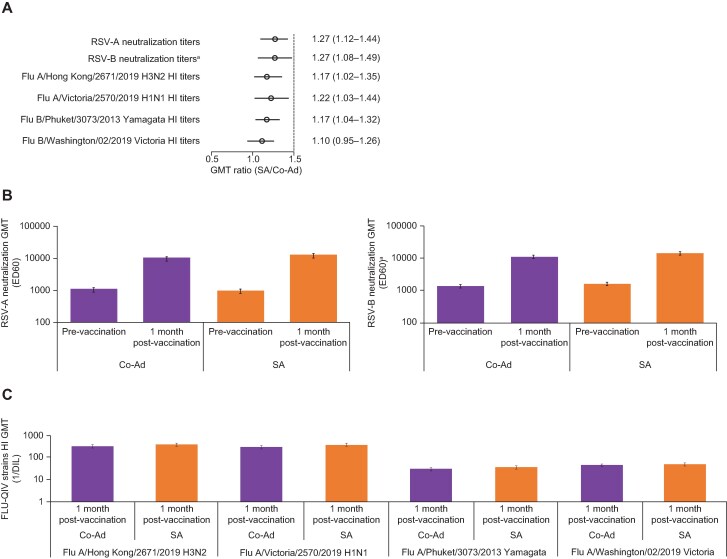
RSV-A and RSV-B neutralization titers and HI titers for each FLU-QIV strain expressed as GMTs and group GMT ratios at pre- and 1 month post-dose—per protocol set. *A*, GMT ratio of SA/Co-Ad RSV-A and RSV-B neutralization and HI titers 1 month post-vaccination. *B*, RSV-A and RSV-B neutralization GMT pre- and 1 month post-vaccination. *C*, HI GMT for each FLU-QIV strain pre- and 1 month post-vaccination. Error bars represent 95% confidence interval. Abbreviations: Co-Ad, co-administration; FLU-QIV, quadrivalent influenza vaccine; GMT, geometric mean titer; HI, hemagglutination inhibition; RSV, respiratory syncytial virus; SA, sequential administration. ^a^Non-confirmatory endpoint.

Both co-primary objectives were met, as the UL of the 95% CI of the GMT ratio for RSV-A and the 4 Flu strain HI titers was ≤1.5 (Figure 3*A*). RSV-A neutralization titers in the Co-Ad group were noninferior compared with the SA group. The GMT ratio was 1.27 (95% CI: 1.12–1.44) between the SA and Co-Ad groups 1 month post-RSVPreF3 OA vaccination (day 31 for Co-Ad group and day 62 for SA group). Similarly, 1 month after the FLU-QIV dose (day 31), the HI titers for each of the FLU vaccine strains in the Co-Ad group were noninferior compared with the SA group. The GMT ratio of FLU A/H3N2, FLU A/H1N1, FLU B/Yamagata, and FLU B/Victoria strains (1/DIL) was 1.17 (95% CI: 1.02–1.35), 1.22 (95% CI: 1.03–1.44), 1.17 (95% CI: 1.04–1.32), and 1.10 (95% CI: 0.95–1.26) between the SA and Co-Ad groups, respectively. The results were consistent when evaluated on the ES (Supplementary Figure 1).

### Secondary Immunogenicity Outcomes

RSV-A neutralization GMTs (95% CI) were 10 060.5 (9126.0–11 090.7) for the Co-Ad and 12 255.0 (11 160.4–13 456.9) for the SA groups 1 month post-RSVPreF3 OA vaccination (Figure 3*B*). RSV-A neutralization titers MGI (95% CI) 1 month post-vaccination over baseline was 9.6 (8.7–10.6) in the Co-Ad group and 12.9 (11.7–14.3) in the SA group. The RSV-B neutralization GMTs were measured in a subset of 425 participants (Figure 3*B*). The GMT ratio of RSV-B neutralization titers between the SA and Co-Ad groups 1 month post-RSVPreF3 OA vaccination was 1.27 (95% CI: 1.08–1.49). At 1 month post-vaccination, RSV-B neutralization titers MGI (95% CI) over baseline was 7.67 (6.8–8.7) in the Co-Ad group and 9.23 (8.0–10.6) in the SA group.

The HI GMTs per FLU-QIV strain 1 month after FLU-QIV were similar for the Co-Ad and SA groups (Figure 3*C*). The HI SCR (HI pre-dose titer <1:10 and a post-dose titer ≥1:40 or a pre-dose titer ≥1:10 and ≥4-fold increase in post-dose titer) met the pre-specified noninferiority criteria (UL of the 2-sided 95% CI on the difference in SCR for SA minus the Co-Ad group ≤10%) for all FLU-QIV strains, except the FLU B/Yamagata strain (UL = 10.1%) (Table 2). Data for HI SPR are in Supplementary Table 2.

**Table 2.** Number and Proportion of Participants With HI Titers Equal to or Above the Cut-Off (SCR)^a^ for Each of the Four Strains at 1 Month Post-Vaccination and Difference Between Groups—Per Protocol Set

	Co-Ad Group (n = 427)			SA Group (n = 410)			SA—Co-Ad	
HI Titers (1/DIL)	n	%	95% CI	n	%	95% CI	Value	95% CI
Flu A/Hong Kong/2671/2019 H3N2	232	54.3	49.5–59.1	233	56.8	51.9–61.7	2.50	−4.2 to 9.2
Flu A/Victoria/2570/2019 H1N1	337	78.9	74.7–82.7	342	83.4	79.5–86.9	4.49	−0.8 to 9.8
Flu B/Phuket/3073/2013 Yamagata	123	28.8	24.6–33.4	134	32.7	28.2–37.5	3.88	−2.4 to 10.1
Flu B/Washington/02/2019 Victoria	152	35.6	31.1–40.3	147	35.9	31.2–40.7	0.26	−6.2 to 6.8

Abbreviations: CI, confidence interval; Co-Ad, co-administration; HI, hemagglutination inhibition; n, number of participants with available results; SA, sequential administration; SCR, seroconversion rate.

^a^HI pre-dose titer <1:10 and a post-dose titer ≥1:40 or a pre-dose titer ≥1:10 and ≥4-fold increase in post-dose titer.

The CBER criteria, in terms of HI SCR and SPR, were met for FLU A strains but not for FLU B strains in both treatment (Co-Ad and SA) and age (60–64 and ≥65 years) groups (Supplementary Figure 2). Regarding the CHMP criteria (Supplementary Figure 3), the MGI for all FLU-QIV strains in both groups was >2.0. The proportion of participants with seroprotective HI titers (≥cut-off value of 1:40 1/DIL) was >60% for the FLU A strains in both groups and for the FLU B/Victoria strain in the SA group. Additionally, the proportion of participants with titers above the SCR was >30% for each of the FLU-QIV strains in both groups, except FLU B/Yamagata in the Co-Ad group.

### Safety and Reactogenicity

**Figure 4.**
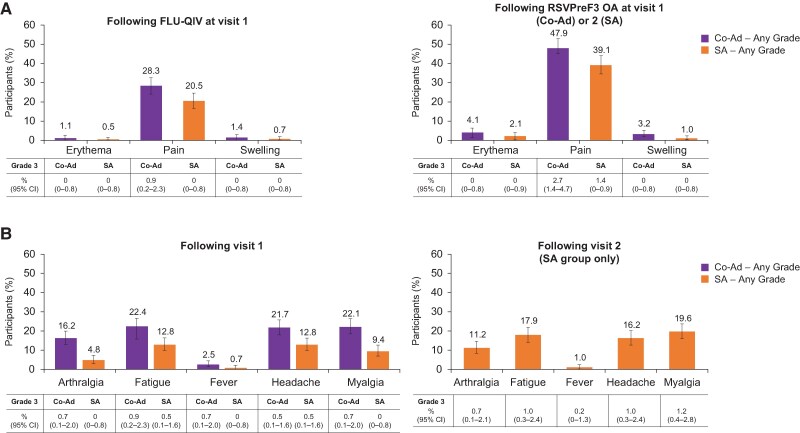
Proportion of participants with solicited (*A*) administration-site and (*B*) systemic AEs—exposed set. Error bars represent 95% confidence interval. Abbreviations: AE, adverse event; Co-Ad, co-administration; FLU-QIV, quadrivalent influenza vaccine; RSVPreF3 OA, AS01_E_-adjuvanted RSV prefusion F protein–based candidate vaccine; SA, sequential administration.

AEs are in Tables 3 and 4. Solicited administration-site AEs were reported in 53.4% in the Co-Ad group and 20.8% in the SA group following FLU-QIV, and in 39.9% in the SA group following RSVPreF3 OA vaccination. Pain was the most frequent AE (FLU-QIV, 28.3% in the Co-Ad group and 20.5% in the SA group; RSVPreF3 OA vaccine, 47.9% in the Co-Ad group and 39.1% in the SA group). Solicited systemic AEs were reported in 40.2% in the Co-Ad group and 24.7% in the SA group following FLU-QIV, and in 34.1% in the SA group following RSVPreF3 OA vaccination (Figure 4). Some participants from each group experienced solicited systemic (Co-Ad, n = 37; SA, n = 39) or administration-site AEs following FLU-QIV (Co-Ad, n = 7; SA, n = 3) and RSVPreF3 OA (Co-Ad, n = 17; SA, n = 21) vaccination, ongoing beyond the 4-day follow-up period (Supplementary Table 3).

**Table 3.** Summary of AEs—Exposed Set

AEs	Co-Ad Group (n = 438)		SA Group (n = 438)	
	n	%	n	%
Solicited administration-site AEs within 4 d following any vaccination	...	...	...	...
Any	234	53.4	195	44.5
Grade 3	13	3.0	6	1.4
Medically attended	1	0.2	0	0
Solicited systemic AEs within 4 d following any dose	…	…	…	…
Any	176	40.2	183	41.8
Grade 3	11	2.5	14	3.2
Medically attended	3	0.7	0	0

	Co-Ad Group (n = 442)		SA Group (n = 443)	
≥1 unsolicited AE within 30 d of any dose	...	...	...	...
Any	83	18.8	105	23.7
Grade 3	13	2.9	15	3.4
Medically attended	35	7.9	49	11.1
≥1 related unsolicited AE within 30 d of any dose	…	…	…	…
Any	26	5.9	15	3.4
Grade 3	1	0.2	2	0.5
≥1 SAE up to study end	15	3.4	20	4.5
≥1 related SAE up to study end	2^a^	0.5^a^	0	0
Any fatal unsolicited AE up to study end	4	0.9	8	1.8
≥1 pIMD up to study end	5^a^	1.1^a^	1	0.2
≥1 related pIMD up to study end	3^a^	0.7^a^	1	0.2

Abbreviations: AE, adverse event; Co-Ad, co-administration; n/%, number/percentage of participants; pIMD, potential immune-mediated disease; SA, sequential administration; SAE, serious adverse event.

^a^Two events of acute disseminated encephalomyelitis, both reported as related SAEs and related pIMDs, were updated by the investigator after the final study analysis to hypoglycemia and dementia in 1 participant and cerebrovascular accident in another participant, with none being related to the investigational vaccines.

**Table 4.** Summary of Solicited AEs and Grade 3 Solicited AEs Within 4 Days Following Each Vaccination—Exposed Set

	Co-Ad Group				SA Group			
			95% CI				95% CI	
	n	%	LL	UL	n	%	LL	UL
Dosing at visit 1								
N	438	…	…	…	438	…	…	…
Any	279	63.7	59.0	68.2	149	34.0	29.6	38.7
Grade 3	19	4.3	2.6	6.7	2	0.5	0.1	1.6
Administration-site	234	53.4	48.6	58.2	91	20.8	17.1	24.9
Grade 3 administration-site	13	3.0	1.6	5.0	0	0	0	0
Systemic	176	40.2	35.6	44.9	108	24.7	20.7	29.0
Grade 3 systemic	11	2.5	1.3	4.4	2	0.5	0.1	1.6
Dosing at visit 2^a^								
N	0	…	…	…	419	…	…	…
Any	…	…	…	…	212	50.6	45.7	55.5
Grade 3	...	…	…	…	16	3.8	2.2	6.1
Administration-site	…	…	…	…	167	39.9	35.1	44.7
Grade 3 administration-site	…	…	…	…	6	1.4	0.5	3.1
Systemic	…	…	…	…	143	34.1	29.6	38.9
Grade 3 systemic	…	…	…	…	13	3.1	1.7	5.2

Abbreviations: AE, adverse event; CI, confidence interval; Co-Ad, co-administration; LL, lower limit; n/%, number/percentage of participants; SA, sequential administration, UL, upper limit.

^a^Participants in the Co-Ad group received both vaccinations at visit 1 and no vaccination was administered at visit 2.

At least 1 unsolicited AE was reported by 83 (18.8%) and 105 (23.7%) participants in the Co-Ad and SA groups, respectively. The most frequent unsolicited AEs were headache (2.3%) and cough (2.0%) in the Co-Ad group, and upper respiratory tract infection (2.3%) and headache (2.0%) in the SA group. The most common related unsolicited AEs were under the higher-level term injection-site reactions (Co-Ad, 1.6%; SA, 1.1%). At least 1 SAE was reported by 15 (3.4%) and 20 (4.5%) participants in the Co-Ad and SA groups, respectively. The most frequent SAE in both groups was confirmed/suspected COVID-19 infection (Co-Ad, n = 4; SA, n = 6). SAEs with a fatal outcome were reported in 4 (0.9%) participants in the Co-Ad group and 8 (1.8%) participants in the SA group. Two SAEs (acute disseminated encephalomyelitis) were initially assessed as related to FLU-QIV by the investigator but were subsequently modified by the investigator to hypoglycemia and dementia in 1 participant and cerebrovascular accident in the second participant, with none being related to the investigational vaccines.

Up to the study end, pIMDs considered as related to the study interventions by the investigator were reported in 2 participants: 1 case of gout in the Co-Ad group which resolved after 3 days and was considered related to FLU-QIV by the investigator, and a second case of gout in the SA group which resolved after 2 days and was considered related to both FLU-QIV and RSVPreF3 OA vaccination.

## DISCUSSION

Routine co-administration of an RSV vaccine with the influenza vaccine could enhance vaccine uptake and benefit the healthcare system by protecting older adults from both infections and limiting the number of required healthcare visits [37, 38]. In our study, the immune response with co-administration of the RSVPreF3 OA vaccine and FLU-QIV was noninferior to the immune response to sequential vaccine administration, in terms of GMT ratios for RSV-A neutralization and HI titers for each FLU-QIV strain.

With respect to the secondary descriptive objective, reference criteria in terms of HI SCR were met for all the FLU strains, except FLU B/Yamagata; this observation was regardless of whether the vaccines were co-administered or administered alone, suggesting that this effect is unlikely to be associated with co-administration.

The mild-to-moderate local and systemic AEs in the Co-Ad group were of a short duration with a low number of grade 3 events. The observed unsolicited AEs, SAEs, and pIMDs were balanced between the groups, and no clustering of events or safety concerns were identified. Co-administration of the RSV vaccine and FLU-QIV was well tolerated, with an acceptable safety profile.

To the best of our knowledge, this is the first phase 3 study to evaluate Co-Ad versus SA for influenza and RSV. The strengths of this study include the sample size and multi-country, randomized controlled design. Limitations include the open-label design and that only humoral immunity was assessed.

A phase 3 randomized trial (NCT04732871) reported strong humoral and cellular immune responses elicited by RSVPreF3 OA, persisting above pre-vaccination levels for at least 6 months post-vaccination [29]. Furthermore, in a phase 3 randomized trial (NCT04886596) conducted in ∼25 000 participants from 17 countries, RSVPreF3 OA demonstrated statistically significant and clinically meaningful efficacy in adults aged ≥60 years; vaccine efficacy against RSV-related lower respiratory tract disease (confirmed by reverse transcription polymerase chain reaction) was 82.6% (95% CI: 57.9–94.1). In this study, RSV-A and RSV-B neutralization titers in the RSVPreF3 OA group increased by a factor of 10.2 and 8.6, respectively, between baseline and 1 month post-vaccination; similar to that observed in our study, whereby RSV-A and RSV-B neutralization titers in the Co-Ad group increased by a factor of 9.5 and 7.7, respectively [31].

In conclusion, this supports co-administration of RSVPreF3 OA and FLU-QIV. Co-administration is well tolerated, with an acceptable safety profile providing a promising opportunity for concomitant vaccination.

## Supplementary Material

ciad786_Supplementary_Data

## Supplementary Data

Supplementary materials are available at *Clinical Infectious Diseases* online. Consisting of data provided by the authors to benefit the reader, the posted materials are not copyedited and are the sole responsibility of the authors, so questions or comments should be addressed to the corresponding author.

## Notes

***Author Contributions.*** Conceptualization: A. L., A. O., D. D., and S. O. K.; Methodology: A. L., A. O., D. D., and S. O. K.; Formal analysis and investigation: A. L., A. O., D. D., S. G., S. O. K., and V. H.; Writing—review and editing: all authors; Resources: C. L., L. N. A. N. M., and R. C.; Supervision: A. O. and V. H.

***Acknowledgments.*** AS01 is a trademark owned by or licensed to the GSK group of companies. The GSK proprietary AS01 adjuvant system contains QS-21 STIMULON adjuvant licensed from Antigenics Inc., a wholly owned subsidiary of Agenus Inc. The authors thank Mohammed Bensellam for contributing to the study conduct and analysis, and Jelena Tica for protocol development. This work was supported by GSK. Medical writing support was provided by Stavroula Bitsi and Alex Coulthard of Apollo, OPEN Health Communications, funded by GSK Biologicals SA, in accordance with Good Publication Practice 3 (GPP) guidelines (www.ismpp.org/gpp-2022).

***Compliance with ethics guidelines.*** This study was conducted in accordance with the ethical principles that have their origins in the Declaration of Helsinki and the principles of Good Clinical Practice, and was approved by all applicable regulatory agencies, investigational center ethics committees, or institutional review boards.

***Data sharing.*** The study report, including the protocol, is available on the GSK Clinical Study Register (https://www.gsk-studyregister.com). Anonymized individual participant data and study documents can be requested for further research from: www.clinicalstudydatarequest.com.
