# Supplementary material for: Immunogenicity, Reactogenicity, and Safety of AS01E-adjuvanted Respiratory Syncytial Virus (RSV) Prefusion F Protein-based Candidate Vaccine (RSVPreF3 OA) When Co-administered With a Seasonal Quadrivalent Influenza Vaccine in Older Adults: Results of a Phase 3, Open-Label, Randomized Controlled Trial
Source: Clin Infect Dis. 2024 Jan 8;83(1):e62–70. doi: 10.1093/cid/ciad786 (PMC13393117; doi:10.1093/cid/ciad786)
Supplement: ciad786_Supplementary_Data [file ciad786_supplementary_data.docx]

**Supplementary Material**

**Supplementary Methods**

**Randomization Methods**

An automated internet-based system, Source data Base for Internet Randomization (SBIR) was used for randomization and for identification of intervention material. The randomization algorithm used a minimization procedure accounting for age (60−69, 70−79, or ≥80 years), center, country, and gender. Minimization factors had equal weight in the minimization algorithm. Once a participant identification number was allocated, the randomization system determined the study group and provided the study intervention number to be used for the first dose. The study intervention number(s) for subsequent dosing were provided by the same automated internet-based system (SBIR).

**Exclusion Criteria**

Participants meeting any of the following criteria were excluded:

- Any confirmed or suspected immunosuppressive or immunodeficient condition resulting from disease or immunosuppressive/cytotoxic therapy
- A history of dementia or a medical condition that moderately or severely impairs cognition
- Recurrent or uncontrolled neurologic disorders or seizures
- Any significant underlying illness that in the opinion of the investigator might prevent the completion of the study
- Known hypersensitivity to any component of the vaccines
- Had received (or were planning to receive) any vaccine other than the study interventions during the period beginning 30 days before the first study intervention administration and ending 30 days after the last study intervention administration (except for COVID-19 vaccines)
- Had received any investigational or non‑registered product (drug, vaccine, or medical device) other than the study interventions during the period beginning 30 days before the first study intervention administration or planned use during the study period
- Were previously vaccinated with a respiratory syncytial virus (RSV) vaccine at any time or received an influenza vaccine in the 6 months preceding the study quadrivalent influenza vaccine (FLU-QIV) administration

**Clinical Laboratory Tests: RSV-A and RSV-B Neutralization Assays**

The serum neutralization assay is a functional assay that measures the ability of serum antibodies to neutralize RSV entry and replication in a host cell line. Virus neutralization was performed by incubating a fixed amount of RSV-A strain (Long, ATCC No. VR-26) or RSV-B strain (18537, ATCC No. VR-1580) with serial dilutions of the test serum. The serum-virus mixture was then transferred onto a monolayer of Vero cells (African Green Monkey, kidney, *Cercopitheus aethiops*, ATCC CCL 81) and incubated for 2 days to allow infection of the Vero cells by non-neutralized virus and the formation of plaques in the cell monolayer. Following a fixation step, RSV-infected cells were detected using a primary antibody directed against RSV (Polyclonal anti-RSV-A/B IgG) and a secondary antibody conjugated to horseradish peroxidase, allowing the visualization of plaques after coloration with TrueBlue peroxidase substrate. Viral plaques were counted using an automated microscope coupled to an image analyzer (Scanlab system with a reading software or equivalent). For each serum dilution, a ratio, expressed as a percentage, was calculated between the number of plaques at each serum dilution and the number of plaques in the virus control wells (no serum added). The serum neutralizing antibody titer was expressed in Estimated Dilution 60 and corresponded to the inverse of the interpolated serum dilution that yielded a 60% reduction in the number of plaques compared with the virus control wells, as described by others [Barbas CF *et al*., *Proc Natl Acad Sci*. 1992;89:10164–10168, Bates JT *et al*., *Virology.* 2014;454–455:139–44]. Secondary standards calibrated against the international reference (NIBSC 16/284) were included in every run to allow conversion into international units.

**Sample Size Calculation**

The enrollment target was 880 participants (1:1 group allocation) to obtain ≥786 evaluable participants (393 in each group) for the evaluation of the primary objectives, assuming approximately 10% of the enrolled participants would not be evaluable. Each objective would be evaluated with a nominal type I error of 2.5%. Considering identical true geometric mean titers in both groups with a common population standard error of 0.45 for the RSV-A neutralization antigen and 0.6 for each of the FLU strains in log_10_ transformed concentration, the study power to meet the co-primary objectives would be ≥93.7%.

| **Supplementary Table 1. Summary of Participant Characteristics – Per Protocol Set** | | | | | | | | |
| --- | --- | --- | --- | --- | --- | --- | --- | --- |
|  | Visit 2 | | | | | | Visit 3 | |
|  | Co-Ad Group  (n = 427) |  | SA Group (n = 410) |  | Total  (N = 837) |  | SA Group  (n = 397) |  |
| Characteristic | Value or n | % | Value or n | % | Value or n | % | Value or n | % |
| Age at first dose |  |  |  |  |  |  |  |  |
| Mean (SD), years | 68.4 (6.9) |  | 68.3 (6.8) |  | 68.4 (6.8) |  | 68.2  (6.7) |  |
|  |  |  |  |  |  |  |  |  |
| Age group, years |  |  |  |  |  |  |  |  |
| ≥65 | 275 | 64.4 | 272 | 66.3 | 547 | 65.4 | 259 | 65.2 |
| ≥70 | 174 | 40.7 | 164 | 40.0 | 338 | 40.4 | 158 | 39.8 |
| ≥80 | 36 | 8.4 | 33 | 8.0 | 69 | 8.2 | 30 | 7.6 |
| 60–69 | 253 | 59.3 | 246 | 60.0 | 499 | 59.6 | 239 | 60.2 |
| 70–79 | 138 | 32.3 | 131 | 32.0 | 269 | 32.1 | 128 | 32.2 |
|  |  |  |  |  |  |  |  |  |
| Country |  |  |  |  |  |  |  |  |
| New Zealand | 141 | 33.0 | 137 | 33.4 | 278 | 33.2 | 124 | 31.2 |
| Panama | 148 | 34.7 | 143 | 34.9 | 291 | 34.8 | 142 | 35.8 |
| South Africa | 138 | 32.3 | 130 | 31.7 | 268 | 32.0 | 131 | 33.0 |
|  |  |  |  |  |  |  |  |  |
| Sex |  |  |  |  |  |  |  |  |
| Male | 205 | 48.0 | 195 | 47.6 | 400 | 47.8 | 189 | 47.6 |
| Female | 222 | 52.0 | 215 | 52.4 | 437 | 52.2 | 208 | 52.4 |
|  |  |  |  |  |  |  |  |  |
| Race |  |  |  |  |  |  |  |  |
| Asian | 4 | 0.9 | 5 | 1.2 | 9 | 1.1 | 4 | 1.0 |
| Black or African American | 71 | 16.6 | 63 | 15.4 | 134 | 16 | 61 | 15.4 |
| Native Hawaiian or other Pacific Islander | 2 | 0.5 | 1 | 0.2 | 3 | 0.4 | 1 | 0.3 |
| White | 132 | 30.9 | 128 | 31.2 | 260 | 31.1 | 115 | 29.0 |
| Māori | 7 | 1.6 | 4 | 1.0 | 11 | 1.3 | 5 | 1.3 |
| Mixed race | 210 | 49.2 | 209 | 51.0 | 419 | 50.1 | 211 | 53.1 |
| Other | 1 | 0.2 | 0 | 0 | 1 | 0.1 | 0 | 0 |

Co-Ad, co-administration; n, number of participants; SA, sequential administration; SD, standard deviation.

|  | **Supplementary Table 2.** **Number and Proportion of Participants With HI Titers Equal to or Above the  Cut-Off (SPR)^a^ for Each of the Four Strains Pre-vaccination and 1-Month Post-vaccination – Per Protocol Set** | | | | | | | |
| --- | --- | --- | --- | --- | --- | --- | --- | --- |
|  | | Timepoint | Co-Ad Group | | | SA Group | | |
| HI titers (1/DIL) | |  | n | % | 95% CI | n | % | 95% CI |
| FLU A/Hong Kong/2671/2019 H3N2 | | Pre  1MPD | 435  427 | 70.8  97.4 | 66.3–75.0  95.4–98.7 | 436  410 | 70.2  97.1 | 65.6–74.4  94.9–98.5  29.9–39.1  90.6–95.6  11.7–18.6  49.2–59.0  19.7–27.9  56.3–66.0 |
| FLU A/Victoria/2570/2019 H1N1 | | Pre  1MPD | 435  427 | 35.4  94.6 | 30.9–40.1  92.0–96.6 | 436  410 | 34.4  93.4 |  |
| FLU B/Phuket/3073/2013 Yamagata | | Pre  1MPD | 435  427 | 14.3  47.8 | 11.1–17.9  43.0–52.6 | 436  410 | 14.9  54.1 |  |
| FLU B/Washington/02/2019 Victoria | | Pre  1MPD | 435  427 | 20.0  59.3 | 16.3–24.1  54.4–64.0 | 436  410 | 23.6  61.2 |  |

^a^HI titer ≥1:40 indicating protection.

CI, confidence interval; Co‑Ad, co-administration; HI, hemagglutination inhibition; MPD, month post-dose; n, number of participants; SA, sequential administration; SPR, seroprotection rate.

| **Supplementary Table 3. Solicited AEs Ongoing Beyond the 4-Day Period Following Vaccination (Per Dose)  – Exposed Set** | | |
| --- | --- | --- |
| AE, n | Co-Ad Group (n = 438) | SA Group (n = 438) |
|  |  |  |
| Systemic AEs |  |  |
| Arthralgia | 9 | 8 |
| Fatigue | 9 | 9 |
| Fever | 1 | 1 |
| Headache | 11 | 13 |
| Myalgia | 7 | 8 |
| Administration-site AEs | |  |
| *Following FLU-QIV dosing at visit 1* | |  |
| Erythema | 0 | 0 |
| Pain | 7 | 3 |
| Swelling | 0 | 0 |
| *Following RSVPreF3 OA dosing at visit 1 (Co-Ad) or visit 2 (SA)* | | |
| Erythema | 0 | 1 |
| Pain | 15 | 19 |
| Swelling | 2 | 1 |

AE, adverse event; Co‑Ad, co-administration; FLU-QIV, quadrivalent influenza vaccine; n, number of events; RSVPreF3 OA, AS01E-adjuvanted RSV prefusion F protein–based candidate vaccine; SA, sequential administration.


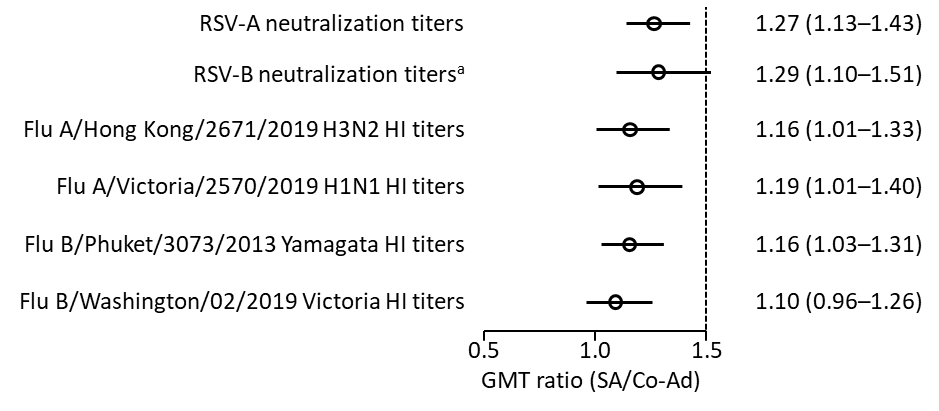


**Supplementary Figure 1.** RSV-A and RSV-B neutralization titers and HI titers for each of the Flu strains expressed as group GMT ratios at pre- and 1 month post-dose – exposed set.
^a^Non-confirmatory endpoint.

Co-Ad, co-administration; GMT, geometric mean titer; HI, hemagglutination inhibition; RSV, respiratory syncytial virus; SA, sequential administration.

**
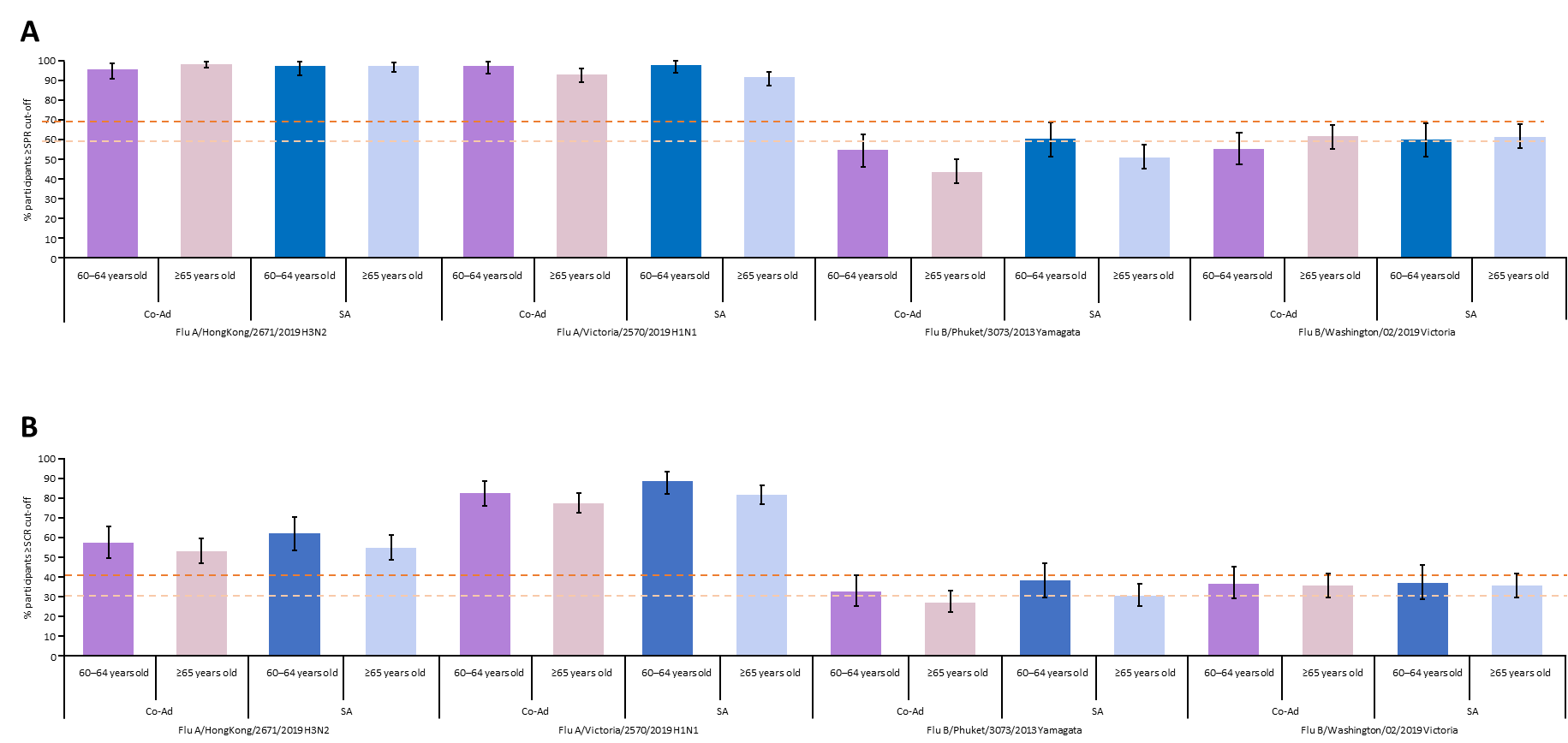
**

**Supplementary Figure 2.** Rate of SPR/SCR by age (CBER criteria). **(A)** SPR and **(B)** SCR for each flu strain. Error bars represent 95% CI. Dark orange and light orange lines represent CBER criteria cut-off values for participants aged 60–64 years and participants aged ≥65 years, respectively.

SPR cut-off value: 1:40 (1/DIL); SCR cut-off value: HI pre-dose titer <1:10 and a post-dose titer ≥1:40 or a pre-dose titer ≥1:10 and ≥4-fold increase in post-dose titer. CBER criteria: The LL of the 95% CI for SPR ≥70% in participants aged 60–64 years or ≥60% in participants aged ≥65 years; The LL of the 95% CI for SCR ≥40% in participants aged 60–64 years or ≥30% in participants aged ≥65 years.
CBER, Center for Biologics Evaluation and Research; CI, confidence interval; Co-Ad, co-administration; HI, hemagglutination inhibition; LL, lower limit;
SA, sequential administration; SCR, seroconversion rate; SPR, seroprotection rate.

**
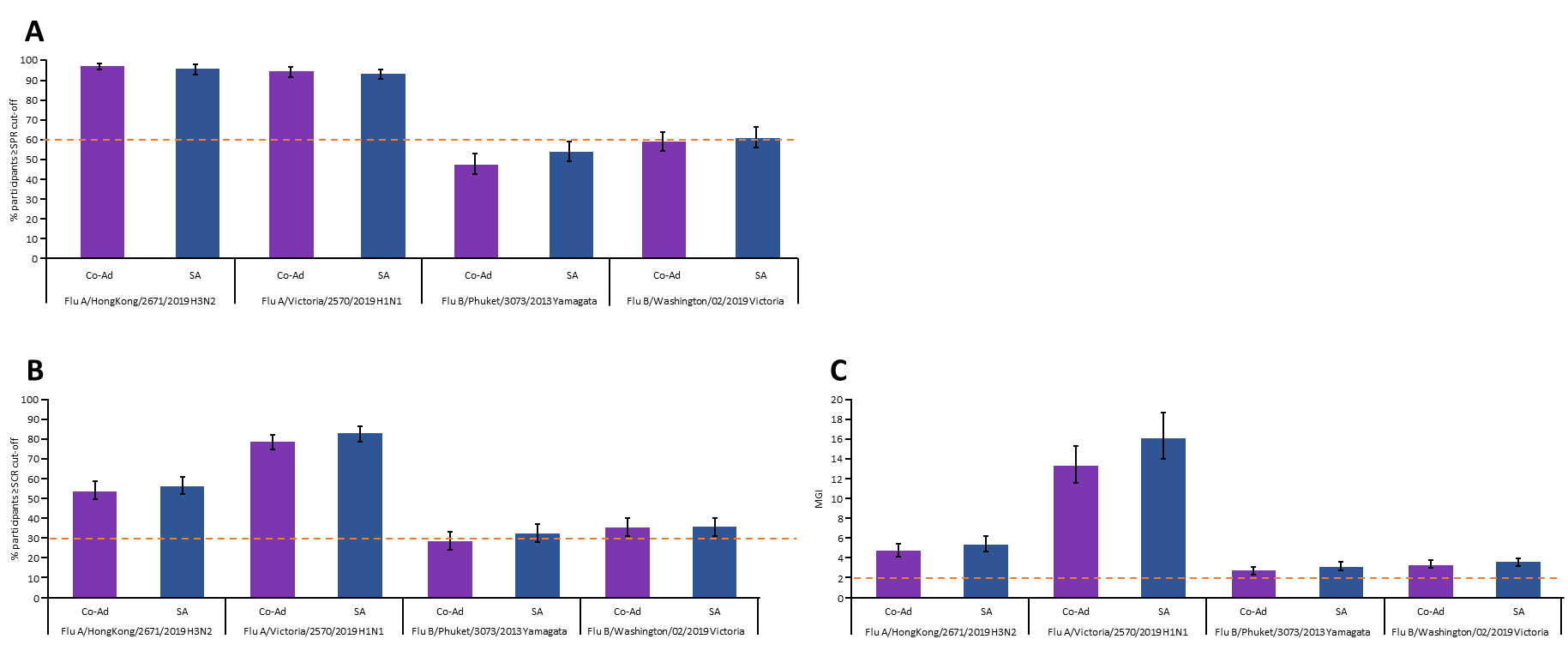
**

**Supplementary Figure 3.** Assessment of CHMP criteria. **(A)** SPR and **(B)** SCR for each flu strain, and **(C)** MGI. Error bars represent
95% CI. Red dashed lines represent CHMP criteria cut-off values. SPR cut-off value: 1:40 (1/DIL); SCR cut-off value: HI pre-dose titer <1:10 and a post-dose titer ≥1:40 or a pre-dose titer ≥1:10 and ≥4-fold increase in post-dose titer. CHMP criteria; ≥1 of the following had to be met: point estimates of SPR >60%, of SCR >30%, or of MGI >2.0.
CHMP, Committee for Medicinal Products for Human Use; CI, confidence interval; Co-Ad, co-administration; HI, hemagglutination inhibition; MGI, mean geometric increase; SA, sequential administration; SCR, seroconversion rate; SPR, seroprotection rate.
